# PPAR*δ* Activation Rescues Pancreatic *β*-Cell Line INS-1E from Palmitate-Induced Endoplasmic Reticulum Stress through Enhanced Fatty Acid Oxidation

**DOI:** 10.1155/2012/680684

**Published:** 2012-06-21

**Authors:** Mingming Cao, Yuzhen Tong, Qingguo Lv, Xiang Chen, Yang Long, Li Jiang, Jun Wan, Yuwei Zhang, Fang Zhang, Nanwei Tong

**Affiliations:** ^1^Division of Endocrinology, West China Hospital, Sichuan University, 37 Guoxuexiang, Chengdu 610041, China; ^2^School of Clinical Medicine, West China Hospital, Sichuan University, 37 Guoxuexiang, Chengdu 610041, China; ^3^Research Laboratory of Endocrine and Metabolic Diseases, West China Hospital, Sichuan University, Chengdu 610041, China

## Abstract

One of the key factors responsible for the development of type 2 diabetes is the loss of functional pancreatic *β* cells. This occurs due to a chronic exposure to a high fatty acid environment. ER stress is caused by an accumulation of irreversible misfold or unfold protein: these trigger the death of functional pancreatic *β* cells. PPAR*δ* is an orphan nuclear receptor. It plays a pivotal role in regulating the metabolism of dietary lipids and fats. However, the correlation between PPAR*δ* of fatty acids and ER stress of pancreatic *β* cells is not quite clear till date. Here, we show that PPAR*δ* attenuates palmitate-induced ER stress of pancreatic *β* cells. On the other hand, PPAR*δ* agonist inhibits both abnormal changes in ER structure and activation of signaling cascade, which is downstream ER stress. Further, we illustrate that PPAR*δ* attenuates palmitate-induced ER stress by promoting fatty acid oxidation through treatment with etomoxir, an inhibitor of fatty acid oxidation. It dramatically abolishes PPAR*δ*-mediated inhibition of ER stress. Finally, we show that PPAR*δ* could protect pancreatic *β* cells from palmitate-induced cell death and dysfunction of insulin secretion. Our work elucidates the protective effect of PPAR*δ* on the fatty-acid-induced toxicity of pancreatic *β* cells.

## 1. Introduction

One of the major risk factors responsible for the development of type 2 diabetes is the loss of functional pancreatic *β* cells: this is usually associated with hyperlipidemia-induced lipotoxicity. The underlying mechanism of this loss of functional pancreatic *β* cells is not fully understood till date [1]. Unlike type 1 diabetes, in the progression of type 2 diabetes, the *β*-cell dysfunction is predominantly associated with persistent hyperglycemia-induced glucotoxicity [2], enhanced level of plasma free fatty acids (FFAs) [3, 4], and increase in circulating cytokines [5] and chronic oxidative stresses [6]. Among these factors, research studies have found that continuous intake of food that is rich in high fatty acids leads to an elevated secretion of insulin from pancreatic *β* cells. Moreover, chronic exposure to an environment with high level of fatty acids results in desensitization and suppression of insulin production, even leading to apoptosis [7]. Therefore, new pharmacological drugs capable of controlling plasma fatty acids are required to treat obesity and type 2 diabetes.

Unlike unsaturated fatty acids, saturated fatty acids, including palmitate, could cause cellular dysfunction and even cell death in pancreatic *β* cells [8]. Recent studies suggested that metabolically generated reactive oxygen species (ROS) is required for fatty-acid-induced apoptosis in pancreatic *β* cells, which could be even aggravated by inhibition of neuronal nitric oxide synthase by using chemical agents [6, 9]. Further, fatty acids synergize with glucose to induce pancreatic *β*-cell apoptosis by activating GSK-3*β* [4]. The specific toxic effects of saturated fatty acids may be related to ceramide formation that an increase in cellular levels of palmitic or stearic acid but not of palmitoleic acid is correlated with de novo synthesis of ceramide, leading to activation of apoptotic signaling pathway [10].

Amounting works have provided evidence linking ER stress with fatty-acids-induced lipotoxicity in *β* cells [8]. The endoplasmic reticulum (ER) is an indispensable organelle for eukaryotic cells. It is used for protein synthesis, folding, and regulating the concentration of calcium ion in cells [11]. ER operates in conjunction with the protein folding pathways. This process is so selective that even relatively minor perturbations in the efficiency of protein folding, termed as ER stress, lead to the rejection of nascent proteins. This ultimately leads to an accumulation or degradation of these proteins. Bip is an abundant multifunctional protein that binds and inhibits several ER stress transducers, including PKR-like ER kinase (PERK), inositol-requiring enzyme 1 (IRE1), and activating transcription factor 6 (ATF6) [12]. Perturbations altering ER homeostasis can accumulate unfolded proteins (UPs). It drives Bip away from these three ER stress transducers, leading to an activation of UPR signaling pathway. The UPR is an integrated intracellular signaling pathway: it triggers transcriptional induction of UPR genes, translational attenuation of global protein synthesis, and ER-associated degradation (ERAD). These provide an adaptive response for survival. Nevertheless, if the protein-folding defect is not corrected, apoptotic signaling pathway mediated by Chop or JNK would be initiated alternatively, thereby leading to cell death [13]. Though the ER stress-initiated prosurvival or proapoptotic signaling cascades were well established, the regulatory network responsible for fatty-acids-mediated ER stress has not been extensively characterized.

Peroxisome proliferator-activated receptors (PPARs) are nuclear receptors. These belong to the steroid receptor superfamily. They play a pivotal role in regulating dietary lipid metabolism and fat storage in mammals [14, 15]. Recent studies have elucidated the protective activity of PPAR*δ* against lipotoxicity. An overexpression of PPAR*δ* significantly reduces the plasma level of FFA as well as the consequent lipotoxicity and improves insulin secretion in pancreatic *β* cells by modulating the oxidation of fatty acids [16–19]. However, the involvement of PPAR*δ* in fatty-acid-mediated ER stress is still not clear.

In this study, we investigated the role of PPAR*δ* in fatty-acid-induced ER stress of pancreatic *β* cells. We proved that PPAR*δ* protects pancreatic *β* cell from palmitate-induced ER stress. Our work leads to a better understanding of the protective effect of PPAR*δ* on fatty-acid-induced toxicity in pancreatic *β* cells.

## 2. Methods

### 2.1. Cell Culture and Reagents

It was cultured in RPMI1640 (Gibco): this medium was supplemented with 10% fetal bovine serum, 10 mM HEPES, 1 mM sodium pyruvate, 2 mM L-glutamine, 50 *μ*M 2-mercaptoethanol, 100 IU/mL penicillin, and 100 *μ*g/mL streptomycin in a humidified atmosphere (5% CO_2_, 95% air) at a temperature of 37°C.

The following three agents were diluted in DMSO: PPAR*δ* agonist GW501516 (Alexis), and PPAR*δ* antagonist GSK0660 (Sigma) and Etomoxir (Sigma) inhibitor of CPT-1, a key enzyme associated with the *β* oxidation of fatty acids. The working concentration of these three agents was 100 nM, 1 *μ*M, and 50 *μ*m, respectively.

### 2.2. Preparation of Palmitate/BSA Solution

10 mM palmitate/10% BSA solution was prepared as follows: 500 mM palmitate was added to 5 mL of 0.1 M NaOH. Then, it was mixed in a water bath that was maintained at 70°C. The resulting solution was mixed with 45 mL of 10% BSA (5 g FFA-free BSA diluted in 45 mL PBS) in a water bath, whose temperature was maintained at 60°C. This solution was stored after it was cooled at room temperature. Cells were treated with 0.5 mM palmitate/0.5% BSA, which was diluted in serum-free RPMI 1640 medium before being used, as previously reported [20–22].

### 2.3. Determination of Insulin Secretion of Pancreatic *β* Cells

INS-1E cells (5 × 10^5^ cells per well) were seeded in standard glucose concentration (11.1 mmol/L) in 6-well dishes. As described by the above procedure, they were treated with corresponding drugs for 48 h. The culture medium was changed every day to ensure that the concentration of the material was kept constant. The preincubation was done for 1 h in a glucose-free Krebs-Ringer bicarbonate buffer (KRBH; 135 mM NaCl, 3.6 mM KCl, 5 mM NaHCO_3_, 0.5 mM NaH_2_PO_4_, 0.5 mM MgCl_2_, 1.5 mM CaCl_2_, 10 mM HEPES, and 0.1% glucose-free and FFA-free BSA, pH 7.4). The cells were washed once in a glucose-free KRBH and subsequently treated with KRBH containing low (2.8 mmol/L) or stimulatory (16.7 mmol/L) glucose concentrations for 1 h. Subsequently, the supernatants were obtained and frozen at −80°C for determining the concentration of insulin. A rat insulin radioimmunoassay kit was used to measure the insulin levels (Linco Research, St Louis, MO, USA). The value of insulin secretion was normalized to the total protein of cells.

### 2.4. Electron Microscopy

The cell pellet was fixed with 0.3% cacodylate-buffered glutaraldehyde (pH 7.4) for 30 min at 4°C. Then, it was centrifuged at 10000 g for 15 min and refixed with 3% cacodylate-buffered glutaraldehyde for 30 min at 4°C. The cell pellet was postfixed in 1% osmium tetroxide (pH 7.4). It was dehydrated in ethanol and embedded in an Epon-Araldite mixture. Ultrathin sections were stained with uranyl acetate and lead citrate. These sections were then examined with a transmission electron microscope (H-600IV; Hitachi, Japan).

### 2.5. Real-Time RT-PCR

Total RNA was isolated using Trizol reagent (Invitrogen) according to the manufacturer's instructions First strand cDNA was reverse-transcribed from 500 ng of total RNA in a final volume of 10 *μ*l using PrimeScript RT reagent Kit (TakaRa, DRR037A), in accordance with the manufacturer's instructions. The primers used were as follows: Bip, 5′-acctttgtggtcctcacctg-3′ and 5′-agctccagttgtggcacttg-3′; ATF4, 5′-cttcagcaaggaggaggtcat-3′ and 5′-ttctcgctctccagaatgtgc-3′; XBP-1s, 5′-cttcagcaaggaggaggtcat-3′ and 5′-ttctcgctctccagaatgtgc-3′; Chop, 5′-ctccagattccagtcagagttc-3′ and 5′-tctcattctcctgctccttctc-3′; CPT-1, 5′-ctgctgtatcgtcgcacattag-3′ and 5′-gttggatggtgtctgtctcttcc-3′; ACO, 5′-agatgtgagtgtgtggcccttac-3′ and 5′-aggaagaccagagtgggagctta-3′; GAPDH, 5′-tatgactctacccacggcaagt-3′ and 5′-atactcagcaccagcatcacc-3′. PCR was performed with SYBR Premix Ex Taq II KIT (TakaRa, DRR081A) in a ABI 7300 real-time PCR system according to manufacturer's instructions.

### 2.6. Western Blotting

Cells were lysed using RIPA lysis buffer (KeyGEN, China), and the supernatant was collected after being subjected to centrifugation at 14,000 g for 10 min at a temperature of 4°C. Protein concentration was quantified by the BCA assay (Pierce, no. 23223). Samples were separated by 10% SDS-PAGE and transferred to PVDF membranes (Amersham Biosciences). The membranes were blocked overnight with PBS containing 0.1% Tween 20 in 5% skimmed milk at 4°C. Then, these were subsequently probed by primary antibodies. Blots were incubated with their respective primary antibodies for 2 h at room temperature. The primary antibodies included JNK (sc-572, Santa Cruz), p-JNK (sc-6254, Santa Cruz), Bip (sc-13968, Santa Cruz), Chop (#2895, Cell Signaling Technology), and BCL-2 (sc-783, Santa Cruz). After washing three times in TBST, the blots were incubated with secondary antibody that was conjugated to horseradish peroxidase for 2 h at room temperature. Blots were visualized using enhanced chemiluminescence reagents (Amersham Pharmacia Biotech, Piscataway, USA). *β*-actin was used as an internal control for validating intracellular proteins. The relative densitometric value of each blot was calculated and analyzed by using Quantity One software (Bio-Rad). 

### 2.7. TUNEL Assay

TUNEL staining was performed using terminal deoxynucleotidyl transferase (Promega Inc., Madison, WI, USA). Cells were fixed in freshly prepared 4% methanol-free formaldehyde solution in PBS (pH 7.4) for 25 minutes at 4°C. These were washed with fresh PBS for 10 minutes at room temperature. These were permeated in 0.2% Triton-100 solution in PBS for another 5 min. After equilibrating for 10 min, the cells were incubated with rTdT buffer and observed through a fluorescence microscope. A nucleus with bright green fluorescence staining was recorded as a TUNEL-positive event. TUNEL-positive cells were counted by using fluorescence microscope (Olympus Optical Co, Hamburg, Germany).

### 2.8. Caspase-3 Activity Assay

The activity of caspase-3 was determined using the Caspase-3 Activity Assay Kit (Beyotime, China). Cells were harvested and washed with PBS two times and then removed. The supernatant material was separated by centrifugation at 10,000 g for 1 min at 4°C. Cells were lysed on ice with lysis buffer for 15 min after being subjected to centrifugation at 16,000 g for 15 min at 4°C. 80 *μ*L analysis buffer, 10 *μ*L caspase-3 substrate (Ac-DEVD-pNA) (2 mM), and 10 *μ*L cell lysate were mixed in a 96-well plate. Then, they were subjected to incubation at 37°C for 2 h. The absorbance of samples was measured using a spectrophotometer at a wavelength of 405 nm.

### 2.9. Data Analysis and Statistics

All the quantitative data were recorded in terms of mean ± SD. Comparisons between two groups were performed by Student's *t*-test. Comparisons among multiple groups were performed by one-way ANOVA.

## 3. Results

### 3.1. PPAR*δ* Attenuates Palmitate-Induced ER Stress in Pancreatic *β* Cells

**Figure 1:**
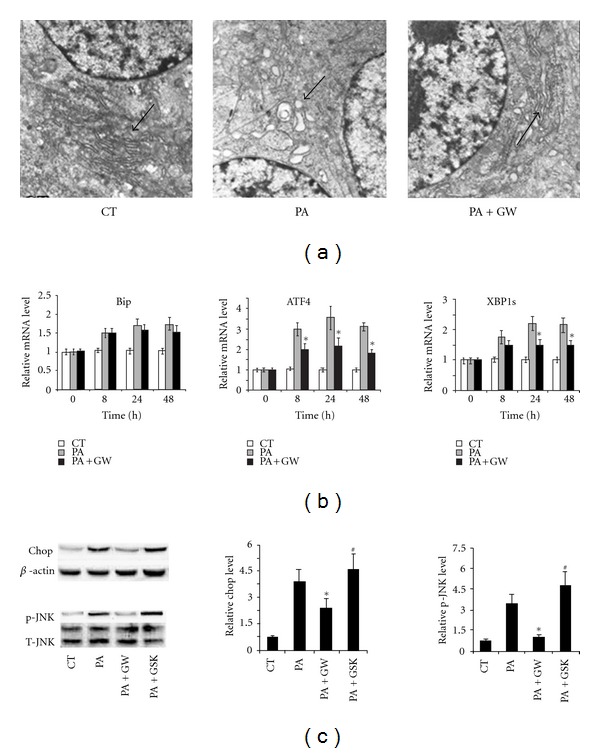
PPAR*δ* attenuates palmitate-induced ER stress in pancreatic *β* cells. INS-1E cells were treated with 0.5 mM palmitate alone or the combination of 0.5 mM palmitate and 100 nM GW501516 for 48 h. (a) Structure of ER was visualized by TEM (×12000). Arrows: the changes in the structure of ER. (b) Expression level of Bip, ATF4, and XBP1s was examined by real-time RT-PCR. INS-1E cells were treated with 0.5 mM palmitate alone, combination of 0.5 mM palmitate and 100 nM GW501516, in combination of 0.5 mM palmitate and 1 *μ*M GSK0660 for 48 h. (c) Expression level of Chop and phosphorylation statues of JNK1 were examined by Western blot. All the data were from three independent experiments (*n* = 3). *P < 0.05: versus palmitate-treated group; ^#^ *P* < 0.05: versus the group treated with the combination of palmitate and GW501516.

We initially examined the role of PPAR*δ* in palmitate-induced ER stress. The structure of ER from INS-1E cells treated by palmitate or the combination of palmitate and GW501516, a chemical activator of PPAR*δ*, was visualized by TEM. As shown, treatment of palmitate caused severe morphologic changes in the ER structure, including abnormal extension and swelling, which was significantly blocked by treating with GW501516 (Figure 1(a)). These results suggest that activation of PPAR*δ* inhibits palmitate-induced ER stress.

It is reported that Bip saves the cell from apoptosis by repressing ER stress [23]. We wondered whether Bip was involved in PPAR*δ*-mediated inhibition of ER stress under palmitate treatment. However, our results indicate that activation of PPAR*δ* had no impact on the expression level of Bip (Figure 1(b)). Notably, we found that palmitate-induced expression of ATF-4 and XBP-1s was attenuated by GW501516 (Figure 1(b)). We had a keen interest in examining the activation status of Chop and JNK, which were downstream effectors of PERK-eIF2-ATF4-Chop and IRE-1-XBP1s-p-JNK signaling pathway, respectively. As expected, GW501516 treatment inhibited either the overexpression of Chop or phosphorylation of JNK (Figure 1(c)). These results indicate that the inhibitory effect of PPAR*δ* on ER stress might be associated with ATF-4 and XBP-1s-mediated signaling pathway.

### 3.2. PPAR*δ* Attenuates Palmitate-Induced ER Stress by Promoting Fatty Acid Oxidation

**Figure 2:**
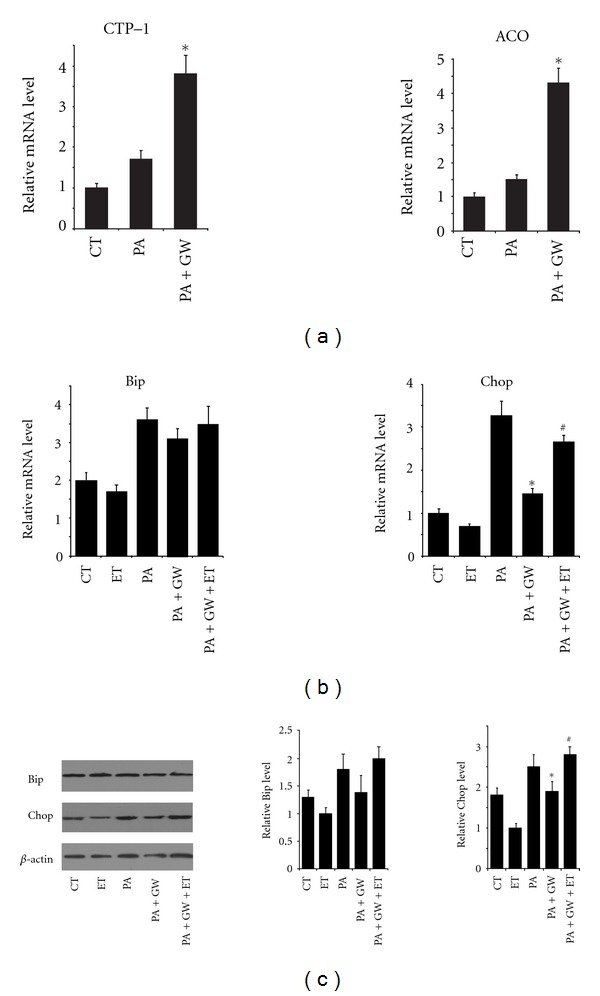
PPAR*δ* attenuates palmitate-induced ER stress by promoting fatty acid oxidation. INS-1E cells were treated with 0.5 mM palmitate alone or the combination of 0.5 mM palmitate and 100 nM GW501516 for 48 h. (a) Expression of CPT-1 and ACO was examined by real-time RT-PCR. INS-1E cells were treated with 0.5 mM palmitate alone, combination of 0.5 mM palmitate and 100 nM GW501516, or combination of 0.5 mM palmitate, 100 nM GW501516, and 50 *μ*m Etomoxir for 48 h. Expression of Bip and Chop was examined by real-time RT-PCR (b) or Western blot (c). All the data were from three independent experiments (*n* = 3). *P < 0.05: versus palmitate-treated group; ^#^ *P* < 0.05: versus the group treated with the combination of palmitate and GW501516.

 PPAR*δ* is an activator of fatty acid oxidation, and fatty acid oxidation can be attenuated with the help of palmitate-induced ER stress [24]. To determine whether lipid oxidation was involved in PPAR*δ*-mediated inhibition of ER stress, the expression of two key enzymes involved in lipid oxidation, CPT-1 and ACO was examined. As shown in Figure 2(a), the expression level of either CPT-1 or ACO was only slightly increased in response to palmitate solo treatment. Strikingly, PPAR*δ* activation notably elevated the expression of both CPT-1 and ACO under palmitate treatment.

To further elucidate if lipid oxidation has a role in PPAR*δ*-mediated inhibition of ER stress, etomoxir, an inhibitor of CPT-1, was used to block fatty acid oxidation. As shown, etomoxir markedly abolished GW501516-mediated inhibition of Chop through treatment of palmitate, at either transcriptional or translational level, though no significant changes were found in the level of Bip (Figures 2(b) and 2(c)). These results indicate that PPAR*δ* attenuates palmitate-induced ER stress by promoting fatty acid oxidation.

### 3.3. PPAR*δ* Protects Pancreatic *β* Cells from Palmitate-Induced Lipotoxicity

**Figure 3:**
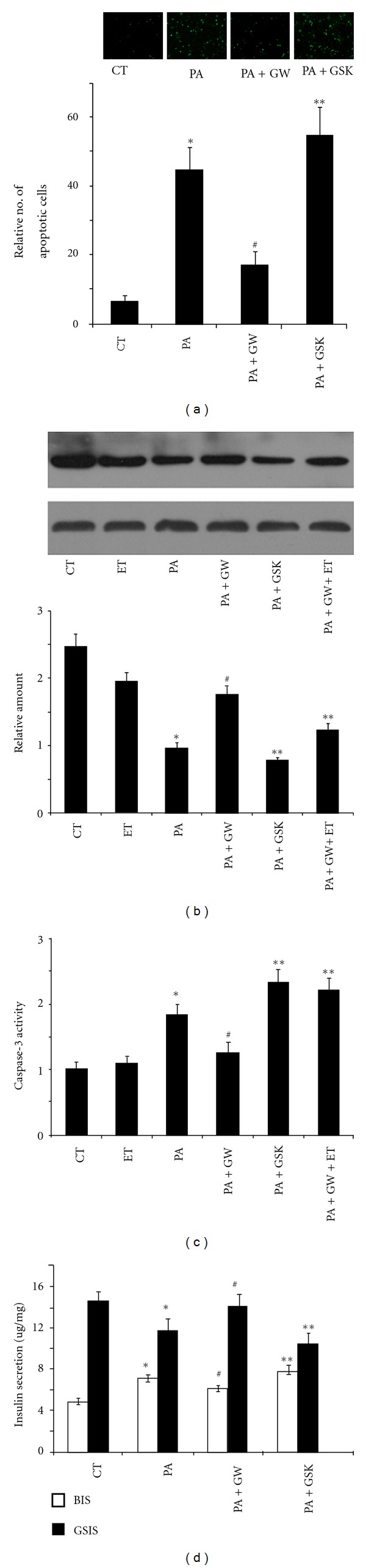
PPAR*δ* protects pancreatic *β* cells from palmitate-induced lipotoxicity. (a) INS-1E cells were treated with 0.5 mM palmitate alone, combination of 0.5 mM palmitate and 100 nM GW501516, combination of 0.5 mM palmitate and 1 *μ*M GSK0660, or combination of 0.5 mM palmitate, 100 nM GW501516, and 50 *μ*m Etomoxir 48 h. Apoptosis was measured by TUNEL assay. (b) Expression of Bcl-2 was examined by Western blot. Upper panal: representative image of immunoblot; pottom panal: statistical analyses of relative densitometric value. (c) INS-1E cells were treated with 0.5 mM palmitate alone, combination of 0.5 mM palmitate, 100 nM GW501516, and 1 *μ*M GSK0660, or combination of 0.5 mM palmitate, 100 nM GW501516, and 50 *μ*m Etomoxir for 48 h. Activity of caspase-3 was examined. (d) INS-1E cells were treated with 0.5 mM palmitate alone, combination of 0.5 mM palmitate, and 100 nM GW501516, or combination of 0.5 mM palmitate and 1 *μ*M GSK0660 for 48 h. BIS and BSIS from INS-1E cells were examined. All the data were from three independent experiments (*n* = 3). *P < 0.05: versus untreated group; ^#^ *P* < 0.05: versus palmitate-treated group; **P < 0.05: versus the group treated with a combination of palmitate and GW501516.

ER stress plays a crucial role in palmitate-induced cell death. Therefore, we examined how PPAR*δ* decreases palmitate-induced lipotoxicity in pancreatic *β* cells. As shown in Figure 3(a), exposure to palmitate-induced apoptosis occurs in INS-1E cells. Notably, the number of apoptotic cells under palmitate treatment was markedly reduced in the presence of GW501516, whereas the inhibition of PPAR*δ* by GSK0660 enhanced palmitate-induced apoptosis. We also examine the impact of PPAR*δ* on the expression level of Bcl-2 and caspase-3, since both Bcl-2 and caspase-3 are crucial factors in apoptotic signaling pathway and abnormal expression of Bcl-2 and caspase-3 is previously reported during ER stress [25, 26]. As a result, activation of PPAR*δ* by GW501516 markedly induced expression of Bcl-2, an anti-apoptotic protein, in response to palmitate. On the other hand, GSK0660-induced PPAR*δ* inhibition slightly reduced the expression of Bcl-2 (Figure 3(b)). Correlatively, inhibition of fatty acid oxidation by etomoxir blocked substantially GW501516-mediated activation of Bcl-2 (Figure 3(b)). These observations were further supported by caspase-3 activity assay (Figure 3(c)). Palmitate-induced activation of caspase-3 was inhibited by GW501516, but it increased by treating with GSK0660 along or in combination with GW501516 and etomoxir (Figure 3(c)).

Further, the role of PPAR*δ* in regulation of insulin secretion was examined by measuring BIS and GSIS. As shown in Figure 3(d), INS-1E cells treated with palmitate exhibited increased BIS but decreased GSIS compared to untreated control. These phenomena were even aggravated in the presence of GSK0660. Notably, activation of PPAR*δ* by GW501516 substantially reversed palmitate-induced BIS elevation and GSIS decrease. These data suggest a protective role of PPAR*δ* against lipotoxicity in pancreatic *β* cells.

## 4. Discussion

PPAR*δ* is one of the members of the nuclear receptor superfamily. Given its effective transcription ability, it could be used in the synthesis of novel drug for treating obesity, hyperlipidemia, and type 2 diabetes [15]. Although the functional role of PPAR*α* and PPAR*γ* in regulating gene transcription and lipid metabolism was extensively documented, the functional role of another family member, namely, PPAR*δ*, was relatively limited. In this study, we illustrated that PPAR*δ* protected pancreatic *β* cells from palmitate-induced ER stress. We showed that exposure to palmitate triggered ER stress in INS-1E cells. Notably, the activation of PPAR*δ* by GW501516 significantly reduces the palmitate-induced extension and swelling of ER. Although, no difference was found in palmitate-induced expression of Bip in presence or absence of PPAR*δ*, PPAR*δ* could abolish the overexpression or phosphorylation of server key signaling transducer downstream ER stress, including ATF4, XBP1s, Chop, and JNK. This is the first paper elucidating the inhibitory effect of PPAR*δ* on palmitate-induced ER stress. Considering the usage of RPMI 1640 medium (11 mM glucose) in our in vitro experimental model that palmitate-induced lipotoxicity might be subsequently aggravated due to the high glucose concentrations, further work is still needed to characterize the palmitate-induced ER stress with or without glucotoxicity.

Previous studies focusing on the role of fatty acid oxidation in palmitate-induced lipotoxicity come to labyrinthic observations. It has long been proposed that enhanced fatty acid oxidation attenuated *β*-cell lipotoxicity by reducing the intracellular level of fatty acids; however, the hydrogen peroxide generated from fatty acids peroxisomal metabolism was also considered as important mediator of *β*-cell toxicity [9, 15]. Moreover, it is reported that stimulation of both lipogenesis and oxidation of fatty acid oxidation protected against palmitate-induced INS-1 cell death, suggesting the protective role of fatty acid metabolism in *β*-cell lipotoxicity [27]. In our data, activation of PPAR*δ* resulted in a similar protective effect of INS-1E cells under ER stress. PPAR*δ* activation elevated fatty acid oxidation in pancreatic *β* cells. These were treated with palmitate that PPAR*δ* agonist significantly upregulated several enzymes of fatty acid oxidation, including CPT-1 and ACO. More importantly, we also proved that increased fatty acid oxidation is indispensable for PPAR*δ*-mediated ER stress inhibition. PPAR*δ*-mediated inhibition of Chop could be restored by inhibitor of fatty acid oxidation with etomoxir, though no significant differences were found in the level of Bip. Our results were consistent with previous works showing that activation of LXR by chemical agents rescued INS-1 cells that underwent palmitate-induced apoptosis [27]. This may be due to the predominant role of either PPAR*δ* or LXR as important activator of fatty acid metabolism.

Prolonged ER stress results in cell death [28]. Having establishing the inhibitory role of PPAR*δ* in palmitate-induced ER stress, in this study, we also estimated the protective role of PPAR*δ* against palmitate-induced lipotoxicity. We showed that palmitate-induced apoptotic cell was markedly reduced in the presence of GW501516 whilst inhibition of PPAR*δ* by GSK0660 enhanced palmitate-induced apoptosis. Moreover, PPAR*δ* could modulate the expression level or/and activity of Bcl-2 and caspase-3, both of which were key factors involved in the apoptotic pathway. Interestingly, we also found that activation of PPAR*δ* restored the insulin secretion by reversing palmitate-induced BIS elevation and GSIS decrease. Our results were in line with previous reports that activation of PPAR*δ* improved both vitality and function of pancreatic *β* cells in either in vitro model or diabetic db/db mice model [1, 29].

In this study, we have investigated the intrinsic link between PPAR*δ* and palmitate-induced lipotoxicity. We have also proved that PPAR*δ* has a protective role in saving pancreatic *β* cells under palmitate treatment as they inhibit the ER stress. Our work might lead to a better understanding of the role of PPAR*δ* as a potential therapeutic target in treating hyperlipidemia and type 2 diabetes.
